# Impact of Electronic Self-Assessment and Self-Care Technology on Adherence to Clinician Recommendations and Self-Management Activity for Cancer Treatment–Related Symptoms: Secondary Analysis of a Randomized Controlled Trial

**DOI:** 10.2196/11395

**Published:** 2019-01-08

**Authors:** Robert Knoerl, Fangxin Hong, Traci Blonquist, Donna Berry

**Affiliations:** 1 Phyllis F. Cantor Center for Research in Nursing and Patient Care Services Dana-Farber Cancer Institute Boston, MA United States; 2 Department of Biostatistics & Computational Biology Dana-Farber Cancer Institute Boston, MA United States

**Keywords:** electronic symptom assessment, self-care, neoplasms, treatment adherence and compliance, quality of life

## Abstract

**Background:**

Patients undergoing cancer treatment experience symptoms that negatively affect their quality of life and adherence to treatment. The early identification and management of treatment-related symptoms are critical to prevent symptom distress due to unmanaged symptoms. However, the early identification and management of treatment-related symptoms are complex as most cancer treatments are delivered on an outpatient basis where patients are granted less face-to-face time with clinicians. The Electronic Symptom Assessment and Self-Care (ESRA-C) promotes participant self-management of treatment-related symptoms by providing participants with communication coaching and symptom self-report, education, and tracking features. While the ESRA-C intervention has been demonstrated to improve symptom distress significantly, little is known as to how the ESRA-C influenced participants’ self-management practices and adherence to clinician recommendations for symptom/quality of life issues (SQIs).

**Objective:**

To compare participant adherence to clinician recommendations and additional self-management strategy use for SQIs between ESRA-C intervention and control (electronic symptom assessment and participant symptom reports alone) group participants. Secondarily, we explored the impact of participant adherence to clinician recommendations and additional self-management strategy use for SQIs on symptom control, symptom management satisfaction, and symptom distress. Lastly, we examined baseline predictors of participant adherence to clinician recommendations and additional self-management strategy use for SQIs.

**Methods:**

This study presents an analysis of a randomized controlled trial. Participants beginning a new chemotherapy or radiotherapy regimen were recruited from oncology outpatient centers and were randomized to receive the ESRA-C intervention or control during treatment. Patients were included in this analysis if they remained on study through the duration of treatment and self-reported at least one bothersome SQI three-to-six weeks after beginning treatment. The Symptom Distress Scale-15 and Self-Management of SQIs Questionnaire were completed two weeks later. Based on Self-Management of SQIs Questionnaire ratings, participants were placed into adherence to clinician recommendations (adhered/did not adhere/did not receive recommendations) and additional self-management strategy use (yes/no) categories.

**Results:**

Most participants were adherent to clinician recommendations (273/370, 73.8%), while fewer used additional self-management strategies for SQIs (182/370, 49.2%). There were no differences in the frequency of participant adherence to clinician recommendations (chi-square test, *P*=.99) or self-management strategy use (chi-square test, *P*=.80) between intervention (n=182) and control treatment groups (n=188). Participants who received clinician recommendations reported the highest treatment satisfaction (n=355, *P*<.001 by analysis of variance; ANOVA), although lowest distress was reported by participants who did not follow clinician recommendations (n=322, *P*=.04 by ANOVA) for top 2 SQIs. Women (n=188) reported greater additional self-management strategy use than men (n=182, *P*=0.03 by chi-square test).

**Conclusions:**

ESRA-C intervention use did not improve participants’ adherence to clinician recommendations or additional self-management strategy use for SQIs in comparison to the control. Future research is needed to determine which factors are important in improving patients’ self-management practices and symptom distress following ESRA-C use.

**Trial Registration:**

ClinicalTrials.gov NCT00852852; https://clinicaltrials.gov/ct2/show/NCT00852852 (Archived by WebCite at http://www.webcitation.org/73rEhNWkU)

## Introduction

Individuals undergoing chemotherapy and radiotherapy for the treatment of hematological and oncological malignancies may experience a variety of distressing symptoms (eg, pain, fatigue, nausea, vomiting, anxiety and depression) [1-3] that negatively affect the quality of life [4,5]. Increased symptom distress due to unmanaged cancer treatment–related symptoms may lead to decreased adherence to cancer treatments [6,7], subsequently increasing the risk of mortality. Thus, the early identification and management of treatment-related symptoms are critical to prevent severe symptom distress due to unmanaged symptoms.

The early identification and management of treatment-related symptoms in individuals undergoing anticancer therapy are complicated by the current norms of cancer treatment delivery. The majority of cancer treatment is now delivered in outpatient settings [8] where patients are granted less clinic time with clinicians to report treatment-related symptoms and review management recommendations than would be possible with inpatient care. Due to decreased face-to-face time with clinicians to review symptoms, patients are expected to seek out and implement strategies to self-manage treatment-related symptoms between clinic visits. Thus, interventions are needed that support patient self-identification and management of treatment-related symptoms during cancer treatment.

Electronic platforms are emerging as promising tools to deliver self-management strategies that aid patients in the assessment and management of treatment-related symptoms during and after the completion of cancer treatment [9-12]. According to a recent conceptual framework of self-management education support for patients with cancer [11], implementation of cancer self-management interventions are thought to improve health outcomes (eg, reduce symptom severity, improve quality of life, lower health care use) by increasing patients’ skill acquisition (eg, disease knowledge, adherence to clinician recommendations, goal setting, self-efficacy, self-monitoring, communication with health care team).” The Electronic Symptom Assessment and Self-Care (ESRA-C) is a symptom assessment and self-management program for remote plus point of care use. The ESRA-C has been demonstrated to mitigate cancer symptom distress [13] in participants receiving chemotherapy and radiation. It is thought that ESRA-C use may decrease cancer symptom distress by enhancing participant- clinician communication about symptom management (eg, participant adherence to clinician recommendations) and participants’ self-management practices. However, it remains unknown how the effect of the ESRA-C intervention on participants’ self-management practices and adherence to clinician recommendations for symptom/quality of life issues (SQIs) contributed to changes in symptom distress, perceived control over symptoms, or treatment satisfaction.

The primary aim of this study was to compare participant adherence to clinician recommendations and additional self-management strategy use for SQIs between individuals randomized to receive either the ESRA-C intervention or control [13]. The secondary aims were to (1) explore the impact of participant adherence to clinician recommendations and additional self-management strategy use for SQIs on perceived control over symptoms, satisfaction with symptom management, and symptom distress and (2) explore baseline characteristics predictive of participant adherence to clinician recommendations and additional self-management strategy use.

## Methods

### Design, Sample, and Setting

This study is an analysis of a secondary objective in a previously conducted randomized controlled trial (RCT) as described at Clinicaltrials.gov NCT00852852 [13], in addition to 2 exploratory analyses. Results of the original RCT revealed that intervention group participants reported significantly lower symptom distress compared to control group participants from baseline to end-of-study (*P*=.02) [13]. Eligible patients in the original RCT were >18 years of age, ambulatory, beginning a new treatment (eg, chemotherapy or radiation) for cancer, and spoke and read English. All recruitment and data-collection procedures occurred at 2 comprehensive cancer centers located in Seattle and Boston. The original RCT was conducted with oversight from the institutional review boards specific to each study site and written informed consent was obtained from all enrolled participants. From the pool of eligible patients in the original RCT (N=752), participants were included in this analysis if they had remained on study through the duration of anticancer therapy and self-reported at least one bothersome SQI 3 to 6 weeks after beginning treatment.

### Procedures

**Figure 1:**
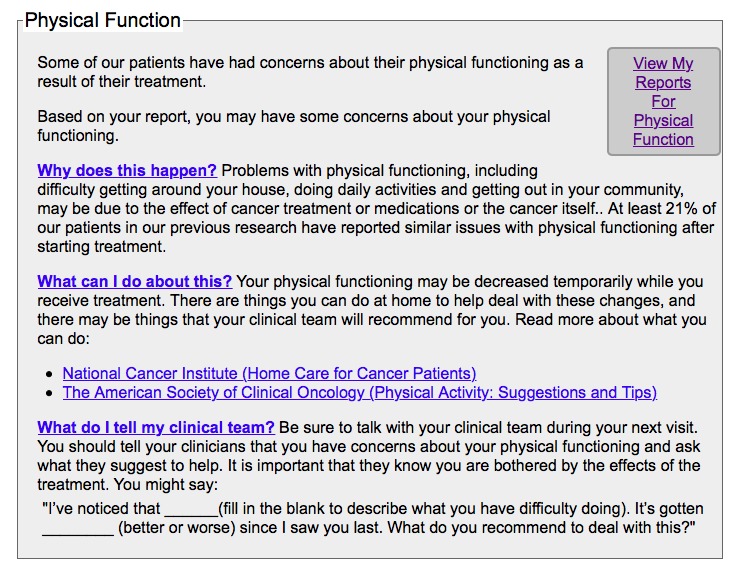
The Electronic Symptom Assessment and Self-Care generated self-management information for physical function.

**Figure 2:**
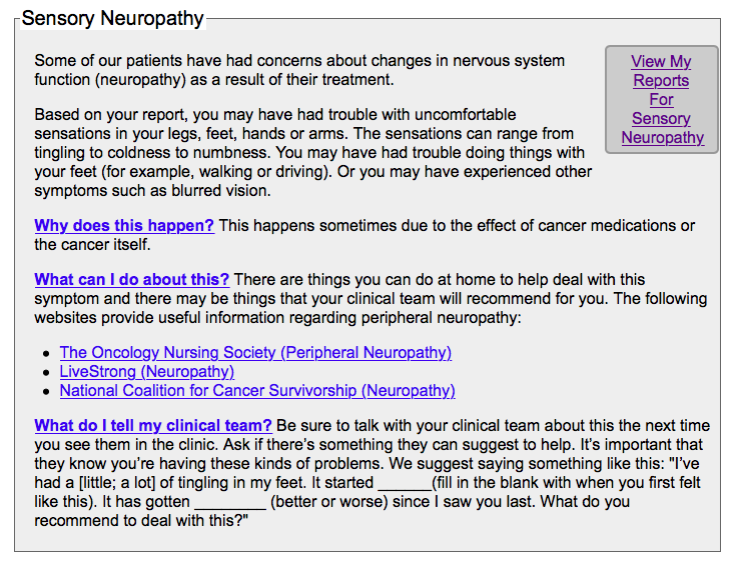
The Electronic Symptom Assessment and Self-Care generated self-management information for sensory neuropathy.

The full procedures of the original RCT were reported elsewhere [13]. Participants were recruited using online and in-person methods at the ambulatory clinics. After signing the informed consent, patients completed the Symptom Distress Scale-15 (SDS-15) [13,14] and a variety of standardized symptom assessment surveys (eg, pain, fatigue, depression, neuropathy, anxiety) before the start of treatment (T1). Next, participants were randomized (1:1 ratio; parallel group) to the ESRA-C intervention or electronic symptom assessment alone. Participants in both groups used the ESRA-C to complete the SDS-15 and the same set of standardized symptom assessment surveys 3 to 6 weeks after beginning treatment (T2) and then 2 weeks later (T3). Following SQI reporting at each time point and regardless of study group assignment, participants’ clinicians (eg, physician, physician assistant or nurse practitioner involved in the care of each participant) received a printed summary of participant symptom reports. Clinicians were oriented to the trial and the use of the participant symptom reports by the principal investigator prior to study initiation, explaining that management of any symptom was at the discretion of the clinician and usual practice. Unique to the intervention arm, the ESRA-C also coached participants with exemplary language regarding how to explain SQI concerns to clinicians, SQI monitoring charts and graphs, and SQI management education. This information was specifically delivered on-screen when an SQI was rated above a predetermined moderate-to-severe threshold, however, participants could access all management strategies through a drop-down menu. Participants were not directly instructed to adhere to the clinician-delivered instruction on SQI management. Examples of the self-management information provided to participants for different SQIs are provided in Figures 1 and 2. Previous analysis revealed that 233/374 (62.3%) intervention group participants in the original trial accessed the ESRA-C intervention (eg, at least two exposures to teaching tips or symptom reports) [15]. Specific to this analysis, participants in both groups reported their top 2 most bothersome SQIs (T2A and T2B) prior to the clinician visit using the ESRA-C at T2 [16] and completed the SDS-15 and Self-Management of SQIs Questionnaire at T3.

### Measures

#### Symptom Distress Scale

The SDS is a 13-item measure in which users rate the frequency and severity of several cancer treatment–related symptoms (eg, nausea, pain, fatigue, insomnia, cough) over the past 7 days [14]. In the primary randomized trial, 2 items (ie, sexual activity and fever/chills) were added to the SDS to form a 15-item version [13]. Items related to problems with sexual activity and fever/chills were added based on informal feedback from patients and clinicians prior to study initiation. Each item is scored from 1 to 5, with total scores ranging from 15 to 75 (higher scores represent worse symptom distress). The 15-item version has demonstrated sufficient internal consistency reliability as evidenced by the Cronbach alpha of .86 in individuals who completed anticancer therapy [13]. The validity of the 15-item SDS has not been tested to date, however, several studies support the concurrent validity of the 13-item version [17].

#### Self-Management of Symptom and Quality of Life Issues Questionnaire

This instrument contains 4 subscales that measure participants’ self-management practices related to their self-reported top 2 bothersome SQIs at T2 (T2A and T2B): (1) adherence to treatment recommendations, (2) self-care activities, (3) perceived control of SQIs, and (4) satisfaction with symptom management. The adherence to treatment recommendations subscale examines the extent to which participants follow clinician recommendations for the management of T2A and T2B. Participants report if they did not receive, did not follow, partly followed, or exactly followed clinician recommendations for the management of T2A and T2B. The self-care activities subscale assesses whether participants use any self-management strategies in addition to clinician recommendations to manage T2A and T2B (yes or no), respectively. The perceived control of SQIs subscale contains 3 questions that measure the degree of control participants perceive over managing T2A and T2B. Each question is scored from 1 to 5 (1 for strongly disagree to 5 for strongly agree), with higher scores representing greater control over SQIs. The Cronbach alpha for the perceived control of SQIs subscale is .74. Lastly, the satisfaction with symptom management subscale examines participants’ satisfaction with T2A and T2B management (self- and clinician-initiated) using a 0 to 10 scale with higher scores representing greater satisfaction. A Cronbach alpha could not be calculated for 3 of the 4 Self-Management of SQIs Questionnaire subscales because they only contain 1 or 2 items that are identical (ie, both items measure T2A and T2B respectively).

### Statistical Analyses

Based on responses to the adherence to treatment recommendations subscale of the Self-Management of SQIs Questionnaire, participants were classified into 3 adherence categories: (1) yes (those who partially/exactly followed recommendations for T2A and T2B, (2) no (did not follow recommendations for T2A and T2B), or (3) no recommendation (participant did not receive management recommendations for T2A and T2B). For participants who reported that no clinician recommendations for T2A and T2B were provided, data from the audio-recorded clinic visit at T2 was used to confirm participant self-report [18]. Participants also were categorized into 2 self-management categories based on responses to the self-care activities subscale: (1) yes (participants who reported using any self-management strategies in addition to clinician recommendations for the management of T2A and T2B) or (2) no (participants who reported no additional self-management strategy use for T2A and T2B).

Baseline characteristics (group assignment, age, gender, working status, employment status, education, ethnicity, cancer type) were described based on adherence to clinician recommendations and additional self-management strategy use categorization. Differences in adherence to clinician recommendations and self-management strategy use between study group and other baseline variables were compared using chi-square tests. Perceived control, satisfaction with symptom management (mean subscale scores were averaged when participants reported a score for both T2A and T2B), and SDS-15 scores were compared among adherence categories (using ANOVA) and additional self-management strategy use categories (by chi-square and two-sample *t* test). The relationship between adherence to clinician recommendations and additional self-management strategy usage were also assessed by a chi-square test. A *P* value of <.05 was considered statistically significant for all comparisons. A complete case analysis approach was used to handle missing data as missing data was minimal. All analyses were conducted using SAS version 9.4.

## Results

### Demographic Characteristics

**Figure 3:**
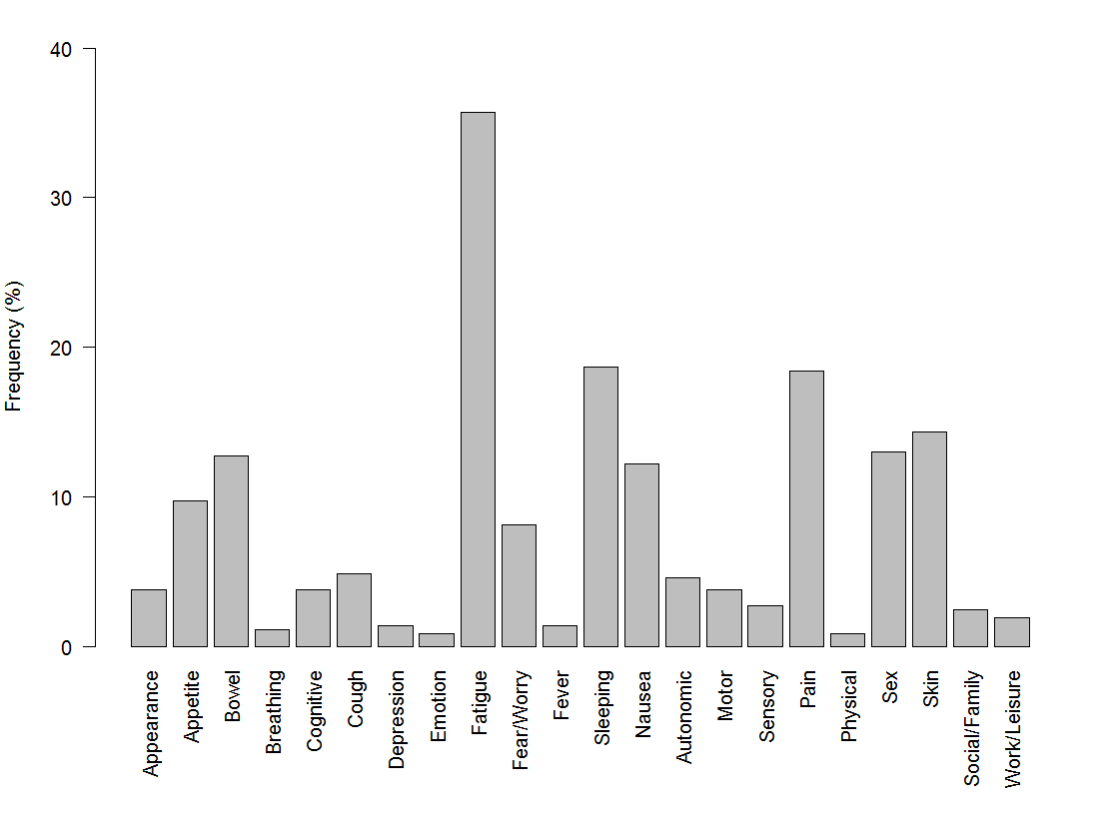
The frequency of the top two bothersome symptom/quality of life issues reported by participants at T2.

Data from 370 (49.2%) participants were available for analysis out of the 752 participants that met eligibility criteria in the RCT. Baseline characteristics of the analyzed sample are summarized by adherence to clinician recommendations and additional self-management strategy use in Table 1. Most of the participants were over the age of 50, currently employed, college educated, under the care of the medical oncology service, and Caucasian. There was a fairly equal number of men and women in the analyzed sample. The median number of days between the T1 and T3 time points was 48 (range 23-159), while the median number of days between T2 and T3 was 15 (range 10-105). Figure 3 describes the frequency of top two bothersome SQIs reported by participants at T2, 3 to 6 weeks after baseline. Fatigue was the most commonly reported bothersome issue, followed by sleep, pain, and skin problems. 

Table 1 describes differences in participant adherence to clinician recommendations and additional self-management strategy use for SQIs among varying baseline characteristics, including treatment assignment. There were no significant differences between assigned groups in the frequency of participant adherence to clinician recommendations (*P*=.99) or additional self-management strategy use (*P*=.80). Women reported significantly greater (*P*=.03) additional self- management strategy use than men, but otherwise, there were no differences in frequency of participant adherence to clinician recommendations or additional self-management strategy use for any other baseline characteristics.

As there were no group differences in participant adherence to clinician recommendations or additional self-management strategy use for SQIs, all subsequent analyses were conducted using the full sample (regardless of the study group). Table 2 describes the frequency of participant adherence to clinician recommendations and additional self-management strategy use for SQIs. Most participants (273/370, 73.8%) partially or completely followed clinician recommendations, while less (182/370, 49.2%) used additional self-management strategies for SQIs. Of the participants that partially or completely followed clinician recommendations, 143/273 (52.4%) also used additional self-management strategies. Conversely, 36/370 (9.7%) participants did not follow clinician recommendations and of those, 23/36 (63.9%) did not use any additional self-management strategies for SQIs. Approximately 13.2% (49/370) of participants reported that they did not receive management recommendations from a clinician. There was no statistically significant association between adherence to clinician recommendations and additional self-management strategy use for T2A and T2B (*P*=.16).

Tables 3 and 4 describe SDS-15, perceived control over SQIs, and satisfaction with symptom management mean scores at T3 based on adherence to clinician recommendations and additional self-management strategy use. Results revealed significant differences in satisfaction with symptom management (*P*<.001) and SDS-15 scores (*P=*.04), and marginally significant differences in perceived control over symptoms (*P=*.10) across adherence categories. Specifically, participants who did not receive clinician recommendations for SQIs reported the lowest satisfaction with symptom management scores. Further, participants who did not follow recommendations reported the lowest symptom distress scores. There were no significant differences in symptom distress, perceived control over symptoms, or satisfaction with symptom management between additional self-management strategy use categories.

**Table 1:** Demographic and cancer treatment–related characteristics by adherence to clinician recommendations and additional self-management activity categorization (N=370).

Characteristics		Adherence, n (%)				Self-management activity, n (%)		
		Missing	No recommendation	No	Yes	Missing	No	Yes
**Study group**								
	Control (n=188)	5 (2.7)	25 (13.3)	18 (9.6)	140 (74.4)	5 (2.7)	88 (46.8)	95 (50.5)
	Intervention (n=182)	7 (3.8)	24 (13.2)	18 (9.9)	133 (73.1)	8 (4.4)	87 (47.8)	87 (47.8)
**Age at baseline**								
	<50 (n=110)	3 (2.7)	14 (12.7)	8 (7.3)	85 (77.3)	3 (2.7)	58 (52.7)	49 (44.6)
	>50 (n=260)	9 (3.4)	35 (13.5)	28 (10.8)	188 (72.3)	10 (3.8)	117 (45.0)	133 (51.2)
**Gender**								
	Men (n=182)	6 (3.3)	22 (12.1)	20 (11.0)	134 (73.6)	7 (3.8)	96 (52.8)	79 (43.4)
	Women (n=188)	6 (3.2)	27 (14.4)	16 (8.5)	139 (73.9)	6 (3.2)	79 (42.0)^a^	103 (54.8)^a^
**Education**								
	Missing (n=1)	0 (0.0)	0 (0.0)	1 (100.0)	0 (0.0)	0 (0.0)	1 (100.0)	0 (0.0)
	<College (n=66)	5 (7.6)	6 (9.1)	8 (12.1)	47 (71.2)	5 (7.6)	31 (46.9)	30 (45.5)
	>College (n=303)	7 (2.3)	43 (14.2)	27 (8.9)	226 (74.6)	8 (2.6)	143 (47.2)	152 (50.2)
**Ethnicity/race**								
	Non-Hispanic white (n=303)	11 (3.6)	41 (13.5)	25 (8.3)	226 (74.6)	13 (4.3)	138 (45.5)	152 (50.2)
	Missing (n=30)	0 (0.0)	3 (10.0)	5 (16.7)	22 (73.3)	0 (0.0)	16 (53.3)	14 (46.7)
	Minority^b^ (n=37)	1 (2.7)	5 (13.5)	6 (16.2)	25 (67.6)	0 (0.0)	21 (56.8)	16 (43.2)
**Clinical service**								
	Medical oncology (n=236)	8 (3.4)	33 (14.0)	22 (9.3)	173 (73.3)	9 (3.8)	113 (47.9)	114 (48.3)
	Radiation oncology (n=134)	4 (3.0)	16 (12.0)	14 (10.4)	100 (74.6)	4 (3.0)	62 (46.3)	68 (50.7)
**Work status**								
	Missing (n=36)	1 (2.8)	3 (8.3)	7 (19.4)	25 (70.0)	2 (5.6)	17 (47.2)	17 (47.2)
	Not working (n=113)	6 (5.3)	12 (10.6)	8 (7.1)	87 (77.0)	6 (5.3)	56 (49.6)	51 (45.1)
	Working (n=221)	5 (2.3)	34 (15.4)	21 (9.5)	161 (72.8)	5 (2.2)	102 (46.2)	114 (51.6)
**Cancer type**								
	Bladder (n=12)	1 (8.3)	3 (25.0)	1 (8.3)	7 (58.4)	1 (8.3)	3 (25.0)	8 (66.7)
	Breast (n=120)	2 (1.6)	23 (19.2)	9 (7.5)	86 (71.7)	3 (2.5)	49 (40.8)	68 (56.7)
	Gastrointestinal (n=74)^c^	5 (6.7)	6 (8.1)	9 (12.2)	54 (73.0)	4 (5.4)	38 (51.4)	32 (43.2)
	Head and neck (n=34)	1 (2.9)	1 (2.9)	1 (2.9)	31 (91.3)	1 (2.9)	18 (53.0)	15 (44.1)
	Prostate (n=63)	1 (1.6)	12 (19.0)	11 (17.5)	39 (61.9)	2 (3.2)	35 (55.5)	26 (41.3)
	Other (n=63)^d^	2 (3.2)	4 (6.4)	5 (7.9)	52 (82.5)	2 (3.2)	31 (49.2)	30 (47.6)
	Unknown (n=4)	0 (0.0)	0 (0.0)	0 (0.0)	4 (100.0)	0 (0.0)	1 (25.0)	3 (75.0)

^a^Statistically significant (*P*<.05) difference between groups.

^b^Hispanic or non-white.

^c^Includes colorectal, esophageal, gastric, pancreatic, and other gastrointestinal cancers.

^d^Includes leukemia, lymphoma, myeloma, renal cell cancer, sarcoma, testicular cancer, and other cancers.

**Table 2:** Relationships between adherence to clinician recommendations and additional self-management strategy use for symptom/quality of life issues (N=370).

Self-management activities	Adherence to clinician recommendation, n (%)				
	Missing	No recommendation given	Did not follow recommendation	Partially or completely follow recommendation	Total
Missing	10 (83.3)^a^	0 (0.0)^a^	0 (0.0)^a^	3 (1.1)^a^	13 (3.5)^c^
No	2 (16.7)^a^	23 (46.9)^a^	23 (63.9)^a^	127 (46.5)^a^	175 (47.3)^c^
Yes	0 (0.0)^a^	26 (53.1)^a^	13 (36.1)^a^	143 (52.4)^a^	182 (49.2)^c^
Total	12 (3.3)^b^	49 (13.2)^b^	36 (9.7)^b^	273 (73.8)^b^	370

^a^The percentage values indicate the frequency of participants within each additional self-management strategy use category out of the total number of participants within each adherence to clinician recommendation category.”

^b^The percentage values indicate the frequency of participants within each adherence to clinician recommendation category out of the total number of enrolled participants.

^c^The percentage values indicate the frequency of participants within each additional self-management strategy use category out of the total number of enrolled participants.

**Table 3:** T3 Self-management of Symptom/Quality of Life Issues Questionnaire and Symptom Distress Scale-15 mean scores by adherence to clinician recommendation categorization (N=370).

Measures	Adherence to clinician recommendation categorization^a^, mean (SD)			*P* value
	None given	Did not follow	Partially/completely followed	
Perceived control (n=355)	2.1 (0.7)	2.4 (0.8)	2.3 (0.7)	.10
Satisfaction with symptom management (n=355)	3.9 (2.5)	6.2 (2.2)	6.2 (2.1)	<.001
SDS-15^b^ (n=322)	28.1 (6.7)	24.9 (6.4)	28.4 (7.6)	.04

^a^As not all participants reported perceived control, satisfaction with symptom management, or symptom distress scale scores, the n for each of these respective measures are smaller than the total N enrolled in the study.

^b^SDS-15: Symptom Distress Scale-15.

**Table 4:** T3 Self-management of Symptom/Quality of Life Issues Questionnaire and Symptom Distress Scale-15 mean scores by additional self-management strategy use categorization (N=370).

Measures	Additional self-management strategy use categorization^a^, mean (SD)			*P* value
	No	Yes		
Perceived control (n=353)	2.3 (0.7)	2.4 (0.7)	.19	
Satisfaction with symptom management (n=354)	6.1 (2.5)	5.7 (2.2)	.18	
SDS-15^a^ (n=322)	27.8 (7.3)	28.3 (7.5)	.56	

^a^As not all participants reported perceived control, satisfaction with symptom management, or symptom distress scale scores, the n for each of these respective measures are smaller than the total N enrolled in the study.

^b^SDS-15: Symptom Distress Scale-15.

## Discussion

### Principal Findings

The results of these analyses revealed no differences in participant adherence to clinician recommendations or additional self-management strategy use between participants randomized to receive the ESRA-C intervention or control. Additionally, participant adherence to clinician recommendations for SQIs, but not additional self-management strategy use, was associated with differences in symptom distress and satisfaction with symptom management ratings. Finally, women were more likely to report additional self-management strategy use than men.

Our findings are consistent with several recently conducted RCTs demonstrating that electronic cancer self-management interventions have no effect on self-management practices such as empowerment [19] or self-efficacy [20]. Similar to the original RCT [13], these trials [19,20] significantly improved symptom distress, but not markers of self-management. These findings call into question what exactly mediates improvements in symptom distress following electronic self-management interventions. A 2017 systematic review [11] identified 8 elements critical to the design of self-management education interventions for cancer: (1) facilitate self-efficacy to manage symptoms, (2) facilitate symptom monitoring, (3) support patient-clinician communication, (4) promote acquisition of problem solving skills, (5) facilitate knowledge and health behavior acquisition via goal setting, (6) garner support from health care team, (7) support patient coaching by trained instructor, and (8) tailor self-management toward individuals’ preferences and treatment plan. However, it is unclear which combination of self-management education elements are associated with improvements in patient outcomes [11]. Future research may be directed toward revising the ESRA-C intervention to address additional core self-management education elements and determine which factors are important in improving self-management practices and symptom distress. The identification of self-management factors that mediate symptom distress improvement following electronic cancer self-management interventions will allow for the tailoring of intervention components known to influence symptom distress.

Results demonstrated that participant adherence to clinician recommendations for SQIs, but not additional self-management strategy use, uniquely affected treatment satisfaction and symptom distress following use of the ESRA-C. Further, there was no significant association between participant adherence to clinician recommendations and additional self-management strategy use for SQIs. Because of the unique effects of participant adherence to clinician recommendations on the tested patient outcomes and the lack of association with additional self-management strategy use, these findings may indicate the relative importance of patient adherence to clinician recommendations for SQIs on patient outcomes. In particular, patient adherence to clinician recommendations may be a result of enhanced patient-clinician communication; if patients are clear on their responsibilities related to symptom management, they may be more likely to adhere to treatment. However, the role of improved patient-clinician communication on patient outcomes is unclear as previous analysis has demonstrated that ESRA-C-induced increases in verbal symptom reporting do not mediate symptom distress improvements (*P*=.41) [18]. Finally, those who adhered to clinician recommendations may not have perceived a need to implement additional self-management strategies.

Nevertheless, strategies to support patient-clinician communication are important, but often missing elements of self-management education for patients with cancer [11]. Recent evidence surrounding the use of patient question prompt lists [21], individualized clinician communication training [22], and recommendations from patient-clinician communication clinical practice guidelines [23] may be used to guide the integration of communication coaching strategies into self-management interventions that target both patients and clinicians.

Participants who did not receive clinician recommendations for SQIs had lower satisfaction with symptom management than participants who received recommendations (regardless of adherence to recommendations). Our findings related to participant satisfaction with symptom management and receipt of clinician recommendations are consistent with recent evidence demonstrating that several clinician-related factors, such as patient-clinician communication about oncology care [24], care coordination [25], and the amount of time spent with the clinician during the outpatient visit [26], predict patients’ satisfaction with oncology care. Additionally, participants who did not follow clinician recommendations for SQIs had lower symptom distress scores than individuals who followed recommendations or did not receive them at all. It is possible that moderate-to-severe symptoms may have improved soon after T2, precluding the need to adhere to recommendations (when surveyed at T3). Alternatively, participants who did not follow clinician recommendations may have experienced lower symptom distress because the T2A and T2B selected were most bothersome, but not the most severe symptoms. Symptom distress refers to the frequency and severity of a symptom [27], whereas bother refers to the relative importance of a symptom (eg, incites feelings of worry) [16]. For example, severe or frequent symptoms may not be as bothersome (eg, effect on sexual activities) as symptoms that are less severe and frequent (eg, fatigue) [16]. Previous research analyzing participants’ selections of T2A and T2B when using the ESRA-C technology revealed that participants did not always select the symptom with the highest SDS-15 score as the most bothersome issue [16]. Due to the small number of participants who did not follow clinician recommendations for SQIs, further research is needed to understand such a finding.

Most baseline characteristics were not predictive of participant adherence to clinician recommendations or additional self-management strategy use for SQIs. However, women were more likely than men to report additional self-management strategies for SQIs. Previous research involving the ESRA-C technology revealed that there were no statistically significant differences in the number of times men or women accessed self-management information within the ESRA-C platform [15]. Thus, due to the lack of differences in ESRA-C exposure between men and women, the observed gender differences in additional self-management strategy use for SQIs may be a result of the tendency for women in the United States to use complementary and alternative medicine strategies for cancer self-management more often than men [28].

### Limitations

Our sample was drawn from English speakers at 2 comprehensive cancer centers and cannot be generalized to other settings or non-English speakers. We were unable to determine the impact of ESRA-C use on the promotion of participant adherence to clinician recommendations or additional self-management strategy use for particular SQIs. The current analyses were likely underpowered as they only used a subset of the full sample from the primary trial. We did not collect information related to participants’ opinions of the self- management information or clinician recommendations they received for bothersome SQIs. It is possible that participants did not use additional self-management strategies or adhere to clinician recommendations for bothersome SQIs because they did not find the self-management information or clinician’s recommendations useful.

### Conclusion

The results of these analyses revealed that there were no differences in the frequency of clinician recommendations or self-management strategy use for SQIs between intervention and control group participants. Additional analyses revealed that participant adherence to clinician recommendations was uniquely associated with differences in symptom management satisfaction and symptom distress scores. Further research is needed to determine how varying components of electronic symptom assessment and management platforms influence participant adherence to clinician recommendations or self-management strategy use for SQIs. Identifying which components of electronic symptom assessment and management platforms influence participant cancer symptom self-management may provide insight as to how the use of electronic symptom assessment and management platforms improve patient-reported outcomes such as symptom distress.

### Practice Implications

Differences in participant adherence to clinician recommendations was a crucial factor in self-reported ratings of symptom management satisfaction and symptom distress. Clinicians must use effective communication (eg, establish goals for care and conversations with patients, gain insight surrounding patient’s understanding of their condition, check for patient’s understanding of information provided) [23] and spend sufficient time with the patient to vigilantly assess patients’ self-reported symptoms as the most severe symptoms may not be the most bothersome. In addition, effective patient-clinician communication about symptom management may increase patient adherence to clinician recommendations. Clinicians also may encourage the use of self-management strategies to supplement recommendations for SQIs to increase patients’ cancer symptom self-management behaviors.

## Abbreviations

ANOVA: analysis of variance
ESRA-C: Electronic Symptom Assessment and Self-Care
RCT: randomized controlled trial
SDS-15: Symptom Distress Scale-15
SQI: symptom/quality of life issue
TI: timepoint 1
T2: timepoint 2
T3: timepoint 3
